# Microglia complement signaling promotes neuronal elimination and normal brain functional connectivity

**DOI:** 10.1093/cercor/bhad313

**Published:** 2023-09-16

**Authors:** Senthilkumar Deivasigamani, Mariya T Miteva, Silvia Natale, Daniel Gutierrez-Barragan, Bernadette Basilico, Silvia Di Angelantonio, Laetitia Weinhard, Dmitry Molotkov, Sukrita Deb, Constantin Pape, Giulia Bolasco, Alberto Galbusera, Hiroki Asari, Alessandro Gozzi, Davide Ragozzino, Cornelius T Gross

**Affiliations:** Epigenetics & Neurobiology Unit, EMBL Rome, European Molecular Biology Laboratory, Via Ramarini 32, 00015 Monterotondo, Italy; Epigenetics & Neurobiology Unit, EMBL Rome, European Molecular Biology Laboratory, Via Ramarini 32, 00015 Monterotondo, Italy; Neuroscience Masters Programme, Sapienza University, Piazza Aldo Moro 1, 00185 Roma, Italy; Epigenetics & Neurobiology Unit, EMBL Rome, European Molecular Biology Laboratory, Via Ramarini 32, 00015 Monterotondo, Italy; Division of Pharmacology, Department of Neuroscience, Reproductive and Odontostomatologic Sciences, School of Medicine, University of Naples Federico II, Via Pansini 5, 80131 Naples, Italy; Functional Neuroimaging Laboratory, Istituto Italiano di Tecnologia, Center for Neuroscience and Cognitive Systems @ UNITN, 38068 Rovereto, Italy; Department of Physiology and Pharmacology, Sapienza University of Rome, 00185 Rome, Italy; Center for Life Nano- & Neuro-Science, Istituto Italiano di Tecnologia, 00161 Rome, Italy; Department of Physiology and Pharmacology, Sapienza University of Rome, 00185 Rome, Italy; Center for Life Nano- & Neuro-Science, Istituto Italiano di Tecnologia, 00161 Rome, Italy; Epigenetics & Neurobiology Unit, EMBL Rome, European Molecular Biology Laboratory, Via Ramarini 32, 00015 Monterotondo, Italy; Epigenetics & Neurobiology Unit, EMBL Rome, European Molecular Biology Laboratory, Via Ramarini 32, 00015 Monterotondo, Italy; Epigenetics & Neurobiology Unit, EMBL Rome, European Molecular Biology Laboratory, Via Ramarini 32, 00015 Monterotondo, Italy; Cell Biology and Biophysics Unit, EMBL Heidelberg, Meyerhofstraße 1, 69117 Heidelberg, Germany; Epigenetics & Neurobiology Unit, EMBL Rome, European Molecular Biology Laboratory, Via Ramarini 32, 00015 Monterotondo, Italy; Functional Neuroimaging Laboratory, Istituto Italiano di Tecnologia, Center for Neuroscience and Cognitive Systems @ UNITN, 38068 Rovereto, Italy; Epigenetics & Neurobiology Unit, EMBL Rome, European Molecular Biology Laboratory, Via Ramarini 32, 00015 Monterotondo, Italy; Functional Neuroimaging Laboratory, Istituto Italiano di Tecnologia, Center for Neuroscience and Cognitive Systems @ UNITN, 38068 Rovereto, Italy; Department of Physiology and Pharmacology, Sapienza University of Rome, 00185 Rome, Italy; Santa Lucia Foundation (IRCCS Fondazione Santa Lucia), Via Ardeatina, 00179 Rome, Italy; Epigenetics & Neurobiology Unit, EMBL Rome, European Molecular Biology Laboratory, Via Ramarini 32, 00015 Monterotondo, Italy

**Keywords:** microglia, complement, neurodevelopment, cell death, cortex

## Abstract

Complement signaling is thought to serve as an opsonization signal to promote the phagocytosis of synapses by microglia. However, while its role in synaptic remodeling has been demonstrated in the retino-thalamic system, it remains unclear whether complement signaling mediates synaptic pruning in the brain more generally. Here we found that mice lacking the Complement receptor 3, the major microglia complement receptor, failed to show a deficit in either synaptic pruning or axon elimination in the developing mouse cortex. Instead, mice lacking Complement receptor 3 exhibited a deficit in the perinatal elimination of neurons in the cortex, a deficit that is associated with increased cortical thickness and enhanced functional connectivity in these regions in adulthood. These data demonstrate a role for complement in promoting neuronal elimination in the developing cortex.

## Introduction

Complement signaling is well documented to be a critical pathway for marking cells for phagocytic engulfment by macrophages in multiple organs across the mammalian body. The complement pathway is triggered by the binding of C1q (Korb and Ahearn 1997; Taylor et al. 2000) to a variety of cell death markers presented on the cell surface of apoptotic cells and the subsequent enzymatic activation of a proteolytic cascade of C3, C4, and C5b-9 (Mevorach et al. 1998; Nauta et al. 2002; Gullstrand et al. 2009). The resulting complex of complement factors serves as an opsonization signal for recognition by macrophages expressing the Complement receptor 3 (CR3; Mevorach et al. 1998). The discovery that complement factors are expressed in the brain and that microglia, the resident phagocytic cells, express CR3 suggested that this pathway was also likely to contribute to neuronal phagocytosis and elimination (Fraser et al. 2010a; Linnartz et al. 2012). The phagocytic elimination of neurons by microglia during brain development is well documented by histological studies in which apoptotic neurons were found surrounded by microglia (Ferrer et al. 1990; Marín-Teva et al. 2004; Peri and Nüsslein-Volhard 2008; Wakselman et al. 2008; Sierra et al. 2010; Abiega et al. 2016) and by studies in which the pharmacological depletion of microglia resulted in an excess of neurons (Marín-Teva et al. 2004).

Nevertheless, evidence emerged showing that mice lacking *CR3* showed deficits in the developmental refinement of retino-thalamic projections (Schafer et al. 2012), which suggested a role for complement in the phagocytic elimination of synapses or axonal branches, rather than whole neurons. Axonal pruning is well documented to occur in interhemispheric cortical projections with over 70% of connections being lost in the first 6 months after birth in primates (LaMantia and Rakic 1990) and similar levels of axonal pruning occur in the postnatal rodent cortex (O’Leary et al. 1981; De León Reyes et al. 2019). Moreover, evidence points to a second wave of synaptic pruning during adolescence (Huttenlocher 1979; Petanjek et al. 2011; Pattwell et al. 2016) that has been implicated in the etiology of schizophrenia (Feinberg 1982; Uhlhaas 2011; Selemon and Zecevic 2015).

However, so far a role for complement signaling in synaptic or axonal pruning outside of the retino-thalamic system lacks strong evidence (Chu et al. 2010; Welsh et al. 2020; Yilmaz et al. 2021). Here we show that cortical structures in mice lacking *CR3* undergo normal adolescent synaptic pruning and perinatal axonal pruning. However, axonal pruning of retino-thalamic projections was significantly reduced. Because axonal pruning in the retino-thalamic pathway is associated with retinal ganglion cell (RGC) death, we hypothesized that complement signaling might exert an effect on neuronal connectivity via its role in promoting microglia-mediated cell elimination. Consistent with this hypothesis, *CR3* mutant mice showed decreased neuronal elimination in the perinatal cortex and an associated increase in cortical cell number and synaptic connectivity in adulthood.

## Materials and methods

### Animals

All mice tested were obtained by internal colonies from the European Molecular biology laboratory. Mice were maintained in a temperature and humidity-controlled condition with food and water provided ad libitum and on a 12-h light–dark cycle (light on at 7:00). *C57BL*/*6J* mice were obtained from local EMBL Rome colonies. The following transgenic mice lines were used: *Thy1*::EGFP-M (Feng et al. 2000; Jackson Laboratory stock 007788) and *Rosa26-CAG::loxP-STOP-loxP-tdTomatoWPRE* (Madisen et al. 2010; Jackson Laboratory stock 007905), CD11b-deficient mice (Coxon et al. 1996; *CR3, C3r, Itgam*; Jackson Laboratory stock 003991), *Emx1::Cre* (Iwasato et al. 2000). Thy1::EGFP animals were bred with *CR3* mice to generate double transgenic mice. Animals homozygous for *Thy1::*EGFP and heterozygous for *CR3* were bred to get animals of the desired genotype. Heterozygote *CR3* animals were bred to obtain WT controls and knockout (KO) animals. For all the experiments, littermate WT and KO were used wherever possible and all the treatments were carried out in random order irrespective of the genotype. Both males and females were used indiscriminately and no exclusion criteria were set a priori. All experiments were performed in accordance 91 with EU Directive 2010/63/EU and under the approval of the EMBL Animal Use Committee 392 and Italian Ministry of Health License 541/2015-PR to CTG. The fMRI experiments were conducted in accordance with EU 86/609/EEC, DL 116, January 1992 and the Guide for the Care and use of Laboratory Animals of the National Institutes of Health. All surgical procedures were performed under anesthesia.

### Statistical analysis

Statistical analysis was performed using either GraphPad 5.0 or SigmaPlot. Plots were obtained with GraphPad Prism 5.0. Each data point refers to an individual animal. The data are presented as mean ± SEM. Spine density analysis, retrograde labeling, quantification of apoptotic cells, cortical thickness, and cell counts were compared using 2-way ANOVA with Tukey’s multiple comparison. Axon density, total axon in optic nerve, quantification of caspase-3 labeled cells were compared with 2-tailed Student’s *t*-test; quantification of microglial engulfment of PSVue labeled cells and *Emx1*::Cre; *RC*::LSL-tomato was compared with Mann–Whitney test and Wilcoxon signed-rank test. Spontaneous excitatory synaptic current (sEPSC) and miniature excitatory synaptic current (mEPSC) data were analyzed with Student’s *t*-test and evoked EPSCs were analyzed with 2-way ANOVA with Bonferroni’s correction. Resting-state fMRI (rsfMRI) data were analyzed by unpaired *t*-test. We used a 95% confidence interval. A *P*-value of < 0.05 was set for rejecting the null hypothesis.

### Data availability

Data were not deposited to any database repositories. All data needed to evaluate the conclusions in the paper are present in the paper and/or the Supplementary Materials. The data supporting the findings are available within the manuscript. Codes used for analysis of neuronal quantitation have been deposited publicly in GitHub.

## Results

### Normal synaptic and axonal pruning in *CR3* knockout mice

**Fig. 1:**
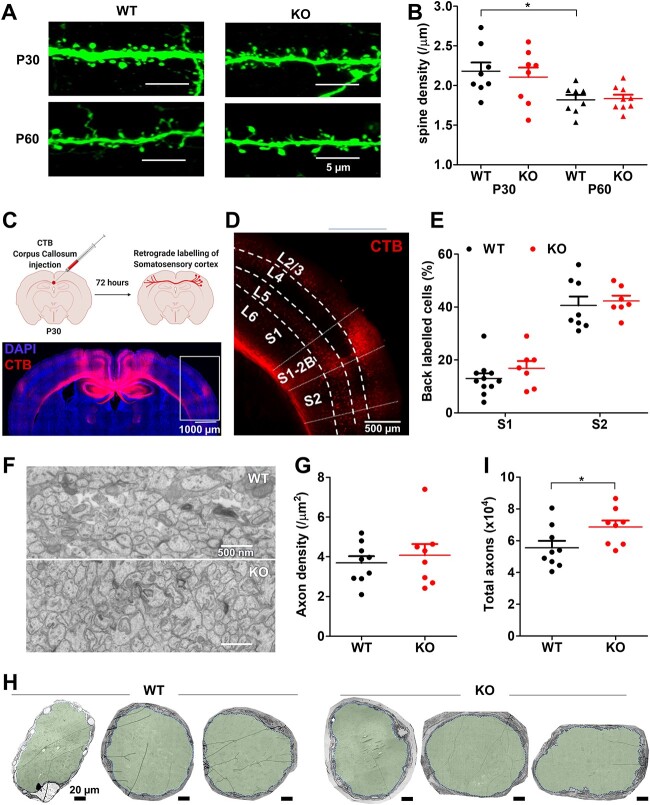
Normal synaptic and axonal pruning but deficient RGC axonal pruning in *CR3* knockout mice. A, B) Pruning of dendritic spines during adolescence was examined in *CR3* knockout mice at P30 and P60. B) Quantification revealed no synaptic pruning deficits in *CR3* knockout mice during adolescence (2-way ANOVA with Tukey’s post hoc test. Main effect of time: *F*[1, 29] = 12.71, *P* = 0.001, main effect of genotype: *F*[1, 29] = 0.11, *P* = 0.737, time × genotype interaction: *F*[1, 29] = 0.25, *P* = 0.620). C) Retrograde labelng was used to examine callosal axon pruning by direct injection of CTB into corpus callosum. D) CTB labeling of S1 and S2 somatosensory cortex regions showing callosally projecting neurons at postnatal day 30. E) Quantification of callosally projecting neurons revealed no differences between wild-type and *CR3* knockout in the S1 and S2 regions of the somatosensory cortex (2-way ANOVA with Tukey’s post hoc test. Main effect of region: *F*[1, 29] = 106.00, *P* < 0.001, main effect of genotype: *F*[1, 29] = 1.15, *P* = 0.292, region × genotype interaction: *F*[1, 29] = 0.18, *P* = 0.672). F, G) Lack of *CR3* does not alter axon density in postnatal day 6 optic nerve (*t*-test, *P* = 0.554). H) *CR3* knockout mice have a marginal increase in optic nerve diameter and I) increase in total axon number (unpaired *t*-test, *P* = 0.045). Each data point refers to an individual animal (mean ± SEM, ^*^P < 0.05, ^*^^*^P < 0.01).

Our initial experiments focused on determining whether mice with deficient complement signaling failed in synaptic pruning as had been shown in the retino-thalamic system (Stevens et al. 2007; Schafer et al. 2012). We chose to study mice lacking the *CR3* (knockout) because this mutant was previously shown to exhibit retino-thalamic pruning deficits and because it is the major complement receptor expressed by microglia (Tremblay et al. 2010; Paolicelli et al. 2011; Filipello et al. 2018). First, we monitored adolescent synaptic pruning by quantifying excitatory spine density in layer 5 neurons of the prelimbic cortex either before (postnatal day 30) or after (postnatal day 60) sexual maturity in the mouse (Fig. 1A). As previously described (Pattwell et al. 2016; Boivin et al. 2018), a significant reduction in spine density was observed in all animals between P30 and P60, but no significant effect of genotype or interaction between time and genotype emerged (Fig. 1A and B). These data suggest that adolescent synaptic pruning of principal cortical neurons proceeds normally in *CR3* knockout mice.

Next, we quantified axonal elimination in interhemispheric cortical connections, a process that involves the pruning of more than 80% of callosal fibers in layer 4 of the primary somatosensory cortex (S1) in the first 3 postnatal weeks of mice (De León Reyes et al. 2019). Local injection of the fluorescent retrograde tracer cholera toxin B (CTB) into fibers of the corpus callosum led to the labeling of contralaterally projecting cell bodies throughout the mouse cortex (Fig. 1C). During the first postnatal week, the vast majority of cell bodies in the layer 4 of S1 were labeled (De León Reyes et al. 2019), whereas at postnatal day 30, <20% of soma showed labeling. This demonstrates the efficient pruning of the majority of interhemispheric axons in this region (Fig. 1D and E). In contrast, the neighboring region (secondary somatosensory cortex; S2) showed less extensive pruning with over 40% of soma being labeled (De León Reyes et al. 2019; Fig. 1D). Quantification of the fraction of labeled cell bodies at postnatal day 30 revealed statistically indistinguishable pruning in *CR3* knockout mice and wild-type littermates in layer 4 of both S1 and S2 cortices (Fig. 1E). These findings indicate that microglia-dependent complement signaling does not have a major role in mediating synaptic or axonal pruning in the developing mouse cortex.

### Deficient RGC axonal pruning in *CR3* knockout mice

Our initial findings suggested that synaptic or axonal pruning deficits in *CR3* knockout mice might be restricted to the retino-thalamic system. To test this possibility, we revisited the pruning phenotype in the visual system of these animals by directly quantifying the perinatal loss of RGC axons. Anatomical studies have shown that during the first postnatal week in rodents approximately half of retinal axons in the optic nerve are eliminated (Lam et al. 1982). Quantification of total axon numbers in electron micrographs of the prechiasmatic optic nerve at postnatal day 6 of *CR3* knockout and wild-type littermates (Figs. 1F and S1b) revealed a significant deficit in axonal pruning in the mutant mice seen as an increased total number of axons without a change in axon density (Figs. 1G–I and S1c). Assuming a 50% loss of axons in the mouse (Lam et al. 1982), these data suggested that microglia-dependent complement signaling is responsible for about one-quarter of axonal pruning in the retino-thalamic system. This deficit in axonal pruning could be a consequence of either incomplete retinal axon or cell elimination.

### Microglia phagocytose apoptotic neurons in early postnatal cortex

However, RGC axonal loss in perinatal development is well documented to be accompanied by RGC death (Potts et al. 1982; Dreher et al. 1983; Perry et al. 1983; Horsburgh and Sefton 1987), opening the possibility that complement signaling in this system may mediate apoptosis. It is well documented that microglia seek out and engulf apoptotic neurons during brain development (Marín-Teva et al. 2004; Wakselman et al. 2008; Cunningham et al. 2013). Two major phases of cortical apoptosis have been described. The first occurs in the subventricular proliferative zone in close connection with cortical neurogenesis and is responsible for the death of a significant fraction of newly born cortical neurons (Blaschke et al. 1996; Thomaidou et al. 1997). Mutations in Caspase 9 that block cell death during this phase of cortical development are associated with macrocephaly, severe cortical malformations, and perinatal lethality (Kuida et al. 1998). Given the viability and grossly unaffected brain morphology and lack of differences in the number of apoptotic cells of between the embryonic *CR3* knockout and wild-type littermate brains (Fig. S2a), we thought it was unlikely that complement proteins would be involved in the death of neuronal precursors. However, a second phase of cortical apoptosis has been described that occurs in the early postnatal period and involves the death of a subset of neurons that have successfully migrated into the cortical plate (Ferrer et al. 1990; Verney et al. 2000; Stankovski et al. 2007; Wong et al. 2018). For reasons that at present remain unclear, this apoptotic phenomenon has been reported to be most frequent in medial cortical regions (Finlay and Slattery 1983; Verney et al. 2000).

**Fig. 2:**
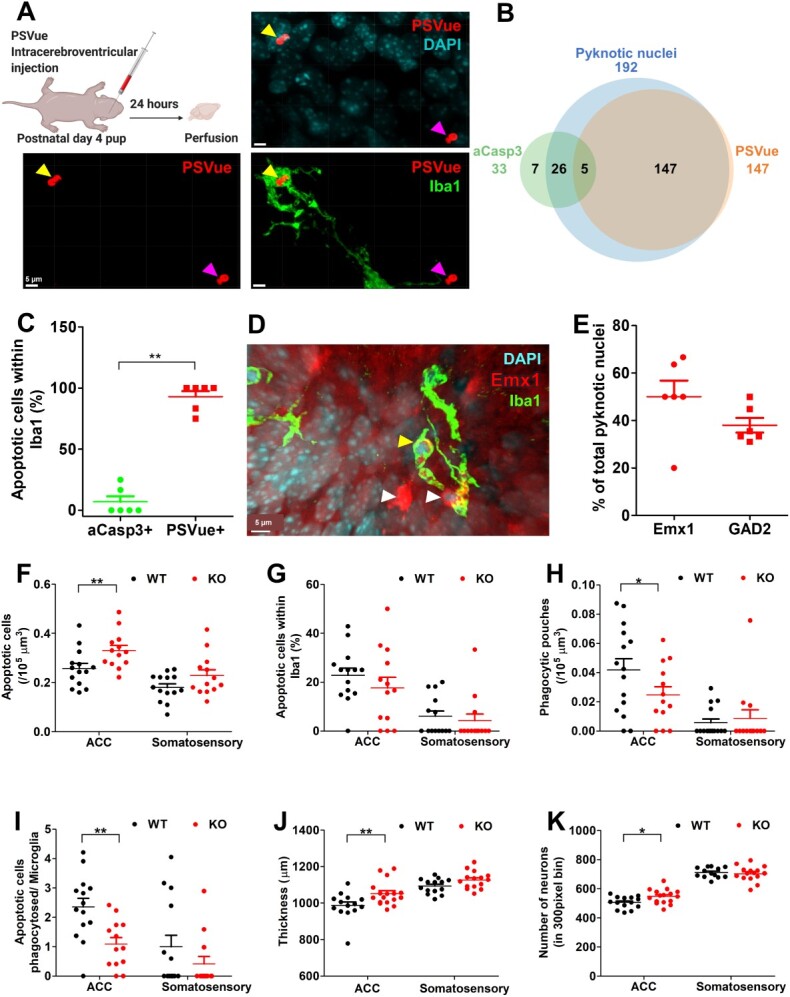
Deficient neuronal elimination in *CR3* knockout mice. A) Apoptotic cells expose PS as labeled with PSVue (magenta arrowhead) and are phagocytosed by microglia (yellow arrowhead). B) Quantification of cell corpse from ACC revealed that the majority of the nuclei are positive for PSVue but not acasp-3. All acasp-3 cells including those devoid of condensed nuclei were considered for quantification. C) PS exposing cells are preferentially phagocytosed by microglia (Mann–Whitney test, *P* = 0.004). D, E) The cell corpse of Emx1 and Gad2 lineage in the ACC as seen by the expression of tdTomato (white arrowhead) is phagocytosed by microglia (yellow arrowhead). F) *CR3*knockout mice have increased density of cell corpse in the ACC but not in the somatosensory cortex (2-way ANOVA with Tukey’s post hoc test. Main effect of region: *F*[1, 50] = 23.19, *P* < 0.001, main effect of genotype: *F*[1, 50] = 10.66, *P* = 0.002, region × genotype interaction: *F*[1, 50] = 0.72, *P* = 0.399). G) A smaller nonsignificant fraction of cell corpse was found engulfed by microglia in the ACC of KO mice when compared with wild type. However, a clear regional difference was observed (2-way ANOVA with Tukey’s post hoc test—main effect of region: *F*[1, 50] = 25.87, *P* < 0.001; main effect of genotype: *F*[1, 50] = 1.06, *P* = 0.307; region × genotype interaction: *F*[1, 50] = 0.19, *P* = 0.665). H, I) *CR3*knockout mice have a reduced number of phagocytic pouches and phagocytosis-apoptosis index in the ACC compared with control littermates (2-way ANOVA with Tukey’s post hoc test—main effect of region: *F*[1, 50] = 49.74, *P* < 0.001; main effect of genotype: *F*[1, 50] = 4.24, *P* = 0.045; region × genotype interaction: *F*[1, 50] = 4.45, *P* = 0.040 and 2-way ANOVA with Tukey’s post hoc test—main effect of region: *F*[1, 50] = 11.22, *P* = 0.001; main effect of genotype: *F*[1, 50] = 9.27, *P* = 0.003; region × genotype interaction: *F*[1, 50] = 1.26, *P* = 0.266). J) Increased ACC cortical thickness is seen in adult KO mice (2-way ANOVA with Tukey’s post hoc test—main effect of region: *F*[1, 56] = 35.44, *P* < 0.001; main effect of genotype: *F*[1, 56] = 10.60, *P* = 0.002; region × genotype interaction: *F*[1, 56] = 1.96, *P* = 0.300). K) Increased number of neurons in the ACC of *CR3* mutant mice (2-way ANOVA with Tukey’s post hoc test—main effect of region: *F*[1, 54] = 35.44, *P* < 0.001; main effect of genotype: *F*[1, 54] = 1.93, *P* = 0.170; region × genotype interaction: *F*[1, 54] = 227.09, *P* < 0.001). Each data point refers to an individual animal (mean ± SEM, ^*^P < 0.05, ^*^^*^P < 0.01).

To determine whether microglia are involved in the phagocytosis of apoptotic neurons during this second phase of cell death, we carried out staining for apoptotic markers and microglia in the anterior cingulate cortex (ACC) of mice at postnatal day 5. A small, but significant number of cells dispersed across cortical layers showed immunostaining for the activated form of the apoptosis-associated factor Caspase 3 (aCasp3+) and in the majority of cases (26/33 cells, 78%) aCasp3+ cells showed condensed, cell corpse (Figs. 2B and S3a). Nevertheless, only a minority of cell corpses expressed aCasp3+ (26/192 cells, 13.5%), suggesting that either caspase activation is not a prerequisite for pyknotic cell death, or that it represents a transient stage in the apoptotic process (Figs. 2B and S3b and e). A significant fraction of pyknotic cells was found to be engulfed by microglia as detected by immunostaining with the marker Iba-1 (Fig. S3c) consistent with the hypothesis that microglia play a major role in the phagocytosis of this type of apoptotic postnatal cortical cell. Compared with aCasp3+ cells, cell corpses labeled with the live fluorescent sensor for extracellular phosphatidyl-serine (PS) modified lipids, PSVue (Eyo et al. 2016; Li et al. 2020; Scott-Hewitt et al. 2020), were preferentially found engulfed by microglia (Figs. 2A and C and S3d–g). These findings indicate that Caspase 3 is activated transiently in cells during the early stages of apoptosis following which these cells progress to nuclear condensation, PS exposure, and identification by microglia for engulfment. To confirm microglia engulfment of cell corpses at this developmental stage in vivo, we used 2-photon live imaging of anesthetized, head-fixed mice at postnatal day 5 with GFP-labeled microglia (*Cx3cr1::GFP*) and pretreated with PSVue. Rare PSVue-positive phagocytic pouches could be observed in cortical layer 2/3 that took 20–60 min to form, and 100–180 min to merge with the microglia soma (Fig. S4a–c). These data provide direct evidence for the phagocytic engulfment of cell corpses by microglia in the early postnatal mouse cortex.

Finally, we examined the identity of the engulfed cell corpse by repeating the co-labeling experiments in mice expressing tomato fluorescent protein in either cortical excitatory neurons (*Emx1*::Cre; *RC*::LSL-tomato; Fig. 2D) or cortical inhibitory neurons (*Gad2*::Cre; *RC*::LSL-tomato). About 60% of the cell corpses were confirmed as deriving from the *Emx1* lineage and 40% of the cell corpses were of the *Gad2* lineage (Fig. 2E). On average, 50% of the condensed cell corpses inside microglia were tomato positive (Fig. S3h). Next, we tested whether cell corpses found inside microglia localized to lysosomes by co-staining with CD68, a lysosomal marker. In the *Emx1* and *Gad2* lines, 77 and 82% of tomato-positive cell corpses, respectively, colocalized with CD68 (Fig. S3i). Lastly, we examined whether the deposition of complement components was associated with pyknotic nuclei. The majority of pyknotic nuclei showed prominent C1q staining when compared with non-pyknotic nuclei (Fig. S4a).

### Deficient neuronal elimination in *CR3* knockout mice

To determine whether complement signaling plays a role in recognizing and engulfing apoptotic cells by microglia, we quantified cell corpses and their engulfment by microglia in the ACC and somatosensory cortex of *CR3* knockout and littermate control mice at postnatal day 5. When compared with controls, the density of cell corpses was significantly increased in *CR3* knockout animals in the ACC, but not in the somatosensory cortex where apoptotic cell density was overall significantly lower (Fig. 2F), consistent with the reported gradient of early postnatal cell death from medial to lateral cortical areas (Verney et al. 2000). A smaller fraction of cell corpses was engulfed by microglia in the ACC of *CR3* mutant mice compared with controls, although this difference was not significant (Fig. 2G). Overall, a significantly larger fraction of pyknotic cells was engulfed by microglia in ACC compared with the somatosensory cortex, further strengthening the idea of a medial-to-lateral gradient (Fig. 2G). We also observed a significant decrease in the number of phagocytic pouches in the ACC of *CR3* mutant mice compared with control littermates, suggesting a reduced phagocytic capacity in the mutants and an overall lower phagocytic capacity in somatosensory cortex compared with ACC (Figs. 2H and S5b). Quantification of a phagocytosis-apoptosis index revealed a highly significant reduction in *CR3* mutant mice compared with controls (Fig. 2I). Together these data suggest that microglia lacking CR3 are less efficient at identifying or engulfing cell corpses and that this process is favored in medial versus lateral cortical structures.

The increased density of cell corpses in *CR3* knockout mice (Fig. 2F) suggests that less efficient microglia phagocytosis might not necessarily be associated with a change in upstream processes that trigger cell death. However, some studies have shown that in the absence of phagocytic engulfment, cellular corpses can be rescued from cell death (Hoeppner et al. 2001; Reddien et al. 2001). Finally, to determine the outcome of an absence of microglia complement signaling on cell survival, we measured cortical thickness and absolute cell numbers in the ACC and somatosensory cortex later in adulthood. Both cortical thickness and the absolute number of neurons were significantly increased in the ACC, but not in the somatosensory cortex in CR3 knockout mice compared with control littermates (Figs. 2J and K and S6a–h). These findings argue for a role of microglia complement signaling as a limiting factor in postnatal cortical neuron elimination.

### Altered synaptic function and functional connectivity in *CR3* knockout mice

**Fig. 3:**
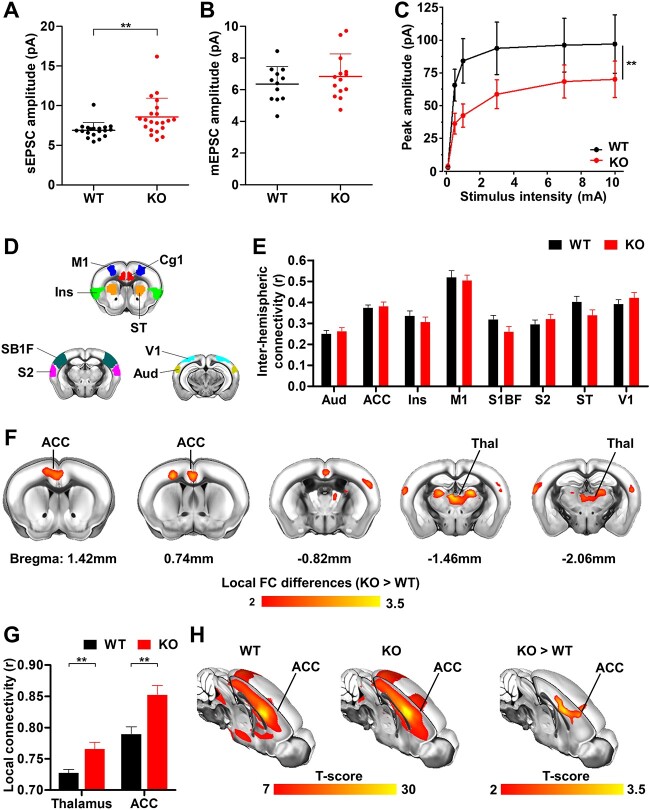
Altered synaptic function and FC in *CR3* knockout mice. A) CR3 mutant mice showed significantly larger sEPSC than wild-type littermates (unpaired *t*-test, *P* = 0.006). B) No difference in amplitude of mEPSC was detected between the *CR3 *mutant mice and wild types (unpaired *t*-test, *P* = 0.66). C) *CR3* mutants exhibit reduced evoked responses than wild-type controls (2-way ANOVA with Bonferroni’s correction—main effect of genotype: *F*[1, 265] = 8.24, *P* = 0.004; main effect of stimulation intensity: *F*[5, 254] = 14.96, *P* < 0.001; stimulation intensity × genotype interaction: *F*[5, 265] = 0.44, *P* = 0.810) each data point refers to an individual cell. D, E) Group-level quantification of interhemispheric FC between pairs of mirror cortical regions shows no alterations in long-range homotopic connections in KO mice. F) Voxel-wise rsfMRI mapping of local connectivity alterations associated with *CR3* mutation (unpaired *t*-test, *P* < 0.05, FWER corrected, cluster-defining threshold *T* = 2). G) Group-level quantification of local connectivity in the thalamus and ACC (unpaired *t*-test, *P* < 0.01). H) Seed-based mapping of the default-mode network spanned by voxel-wise connectivity to the ACC, and group-level differences showing the local effect of the mutation. Each data point refers to an individual animal (mean ± SEM, ^*^P < 0.05, ^*^^*^P < 0.01).

Next, we studied whether the lack of microglia complement signaling during development has any long-term consequence on neural connectivity and synaptic function. First, we quantified spontaneous excitatory synaptic responses of principal neurons in ex vivo hippocampal slices. A significant increase in sEPSC amplitude, but not frequency was observed in *CR3* knockout neurons compared with those from littermate controls (Figs. 3A and S7a–d). However, no significant difference in the amplitude or frequency of mEPSC was detected between genotypes (Figs. 3B and S7a and e–g), although an increase in the relative difference in amplitude between spontaneous and miniature events (Δ amplitude = sEPSC − mEPSC) was found in *CR3* knockouts when compared with controls (Fig. S6h), suggesting increased synaptic multiplicity. In contrast, excitatory synaptic responses to local extracellular electrical stimulation were significantly reduced in *CR3* knockout neurons compared with those from control animals (Figs. 3C and S7i). This apparent discrepancy suggests that while synaptic multiplicity is enhanced in a small subset of active neural connections, on average synaptic strength is reduced in the absence of complement signaling in microglia. In line with our findings from the prefrontal cortex, *CR3* knockout mice did not exhibit any difference in spine density, bouton density, or bouton size in hippocampal pyramidal neurons across development (Fig. S8a–e).

Finally, we used rsfMRI to map interareal functional connectivity (FC) in *CR3* mutants and control littermates. This allowed us to quantify synchronized fluctuations in spontaneous fMRI signals (Power et al. 2014; Gozzi and Schwarz 2016), hence providing an indirect index of communication across brain regions. Importantly, rsfMRI FC is tightly constrained by the underlying anatomical organization and is thereby commonly used to probe the integrity and strength of underlying axonal connections (Coletta et al. 2020). Quantification of rsfMRI FC in interhemispheric region pairs did not reveal any significant difference in CR3 knockout mice compared with control littermates (Fig. 3E). The absence of interhemispheric connectivity changes confirmed our anatomical findings showing a lack of impact of microglia complement signaling on interhemispheric cortical axon pruning (Fig. 1C–E).

We next investigated whether FC could be altered on a finer anatomical scale. For this purpose, we constrained rsfMRI FC mapping to a small 6-voxel radius, corresponding to ~600 μm in a plane—a measure we refer to as local functional connectivity (LFC; Liska et al. 2018; Pagani et al. 2019). Interestingly, KO mice showed foci of increased LFC in midline cortical structures centered around the ACC, as well as in dorsal thalamic regions (mediodorsal thalamus—MD), 2 brain areas known to be highly anatomically interconnected (Fig. 3F and G). To rule out the possibility that these focal LFC differences would indirectly reflect alterations in the long-range connectivity of the corresponding network systems (Gutierrez-Barragan et al. 2022) we measured their long-range connectivity using a seed-based probing of the ACC (Whitesell et al. 2020). Consistent with our previous findings, the observed between-group connectivity differences were focal and short-ranged (Fig. 3H). This finding is consistent with a long-term impact of microglia complement signaling that is specifically targeted to dorsal midline brain regions and that primarily affects microscale anatomy.

## Discussion

The role of complement signaling in synaptic pruning beyond the retino-thalamic system remains unclear. This question gained increased importance with the discovery that common copy number variants in complement factor *C4* showed a dose-dependent association with risk for schizophrenia in human populations (Sekar et al. 2016; Kamitaki et al. 2020; Yilmaz et al. 2021). Recent studies have found that overexpression of C4 in the developing mouse cortex impacts spine density and function (Comer et al. 2020; Yilmaz et al. 2021). However, loss of function mutations in the mouse *C4* gene did not show a phenotype in these studies (Yilmaz et al. 2021), leaving open the question of whether complement mediates cortical pruning in the undisturbed animal. Here we confirmed that mice lacking the microglia complement receptor *CR3* do not show alterations in synaptic or axonal pruning, but instead show deficits in the elimination of apoptotic neurons and that this has a long-term impact on brain FC in a manner that selectively affects Schizophrenia-associated brain regions.

Both the magnitude and direction of our finding of a significant increase in RGC axons in *CR3* knockout mice at postnatal day 6 (Fig. 1F–H) are consistent with earlier data showing an increase in binocular retino-thalamic innervation at the same time point (Schafer et al. 2012). We interpret our data as evidence that the thalamic phenotype of these mice may in part be a secondary consequence of a deficit in the elimination of exuberant retino-thalamic axons, although our study did not carry out measurements of individual axon arbors to test the extent of this relationship. Moreover, as axon elimination in the retino-thalamic system is driven by extensive RGC death (Dreher et al. 1983; Perry et al. 1983), we hypothesize that a critical function of complement signaling in the developing eye is to promote microglia-mediated neuronal cell elimination. This hypothesis is supported by a recent study in which *CR3* (Anderson et al. 2019) knockout mice were shown to have an excess of retina ganglion cells of a magnitude matching our results (Fig. 1F–H). Our findings open the possibility that the synaptic pruning deficits reported earlier in the retino-thalamic system of complement cascade mutants could be at least in part the result of deficits in the elimination of apoptotic RGC.

The removal of cells destined for death and those exposing PS is typically mediated by macrophage phagocytosis and molecules like progranulin have been shown to inhibit the clearance of cells expressing PS. Alternatively, phagocytosis can execute the death of viable cells that reversibly expose PS through phagoptosis (Brown and Neher 2012; Fricker et al. 2012; Neher et al. 2014). In the developing cerebellum, about 60% of dying neuronal cells were contacted by microglial processes. Elimination of microglial cells resulted in increased survival of Purkinje cells suggesting that microglia actively promote cell death in the cerebellum, at least in slice cultures (Marín-Teva et al. 2004). Similarly, in the developing hippocampus, microglia contact and engulf dying cells. Eliminating DAP12 or CR3 genetically or through antibody depletion resulted in reduced apoptosis of hippocampal neurons as estimated by aCasp3 staining (Wakselman et al. 2008). These studies suggest that microglial phagoptosis can promote cell death in the developing brain. We have not examined if phagoptosis occurs in the neonatal cortex. However, we do not see any change in the levels of aCasp3 cells between wild-type and *CR3* knockout mice, suggesting that the absence of CR3 signaling does not promote cell death in the cortex (Fig. S5e).

The relatively modest excess of neurons we see in *CR3* knockout mice appears to be restricted to medial regions of the cortex (Fig. 2J and K) suggesting that they may be the result of a selective deficit in early postnatal cortical programmed cell death that has been shown to occur with a medial to the lateral gradient. This hypothesis is supported by the reduced number of cell corpses found engulfed by microglia in layers 2/3 and 5 of the ACC, but not the somatosensory cortex at postnatal day 5 (Fig. 2G) and a concomitant overall increase in pyknotic cells at this time point (Fig. 2F). It should be pointed out, however, that the engulfment of pyknotic cells by microglia appears to be only partially impaired in *CR3* knockout mice, a finding that suggests that complement signaling is only one of multiple pathways promoting microglial phagocytosis. Complement deposition preceding microglial phagocytosis has been observed in both apoptotic and newborn cells (Fraser et al. 2010b; VanRyzin et al. 2019). Our data are potentially consistent with those of at least one other study that reported a deficit in aCasp3+ cells in the early postnatal hippocampus of *CR3* knockout mice at this stage (Wakselman et al. 2008). Unlike in this study, however, we observed only a mild, nonsignificant decrease in aCasp3+ cell number in *CR3* knockout mice (Fig. S5d) and an increase in cell corpses and decrease in their engulfment by microglia (Fig. 2F). This phenotypic discrepancy could be the result of the different regions examined or could it be a compensatory response to decreased phagocytosis of apoptotic cells in the mutants.

Our observation of anatomical and functional imaging phenotypes in *CR3* knockout mice that were restricted to the anterior dorsal medial cortex (Figs. 2F, J and K and 3G and H) are consistent with the reported gradient of early postnatal neuronal apoptosis along the medial-to-lateral axis (Fig. 2F; Finlay and Slattery 1983; Verney et al. 2000). We noted that despite similar densities of microglia in ACC and somatosensory cortex (Fig. S5c), a significantly smaller fraction of cell corpses was found engulfed by microglia in the more lateral region (Fig. 2G) suggesting that the gradient may be the consequence of a difference in the phagocytic capacity of microglia. It is striking that the regions of the cortex most affected in the complement mutant are those shown by unbiased clustering analysis of rodent anatomical tract-tracing studies to be hubs that support executive function, planning, and self-awareness (Swanson et al. 2017). It remains to be determined whether this gradient is relevant for the link between copy number variation in complement factor *C4* and risk for Schizophrenia (Sekar et al. 2016). Although we cannot at present be certain that the changes in cortical thickness (Fig. 2J and K), synaptic function (Fig. 3A–C), and functional imaging (Fig. 3G and H) observed in adult *CR3* knockout mice depend entirely on deficits in early postnatal cell elimination, the matching medial-to-lateral gradients of postnatal microglia phagocytosis (Fig. 2G), cortical thickness, and functional imaging phenotypes suggest that they may be causally related. rsfMRI FC mapping showed the presence of preserved long-range connectivity alterations but locally increased rsfMRI synchronization in brain regions characterized by deficient perinatal neuronal elimination. Owing to the linear relationship that exists between rsfMRI connectivity strength and underlying axonal density (Coletta et al. 2020), it is tempting to speculate that the observed increased local connectivity may indirectly reflect increased neuronal numbers observed at the microscale in these regions. This interpretation would be consistent with previous observations of reduced local fMRI connectivity in animal models characterized by reduced cortical thickness (Liska et al. 2018; Pagani et al. 2019). In light of the current controversy of the correlates of fMRI connectivity (Rocchi et al. 2022) this interpretation, however, remains tentative and requires further physiological corroboration. It is important to note that the increase in local connectivity did not translate into changes in local field potential in any of the frequency bands (Fig. S9b) and neither did we observe any signs of spontaneous seizures as reported in C1q mutants (Fig. S9a; Chu et al. 2010). Critically, our data argue that the increase in cortical thickness and neuronal number in the adult ACC is a direct consequence of a failure of microglia lacking CR3 to efficiently engulf apoptotic cells in this structure during the early postnatal period. We acknowledge that we have not used stereology-based quantification to estimate neuronal numbers and thus cannot rule out that subtle genotype-dependent changes in the size of neurons could have biased our comparisons. However, we consider it unlikely that deficits in complement signaling would consistently affect nuclear size. A causal link between increased neuronal number and failure of microglial engulfment in *CR3* knockouts requires that in the absence of microglia engulfment apoptotic neurons can reverse their nuclear condensation phenotype and survive to adulthood. Such a reversal of commitment to apoptosis has been described in cultured cells and can include the transient expression of activated caspases, for example (Geske et al. 2001; Tang et al. 2012; Sun et al. 2017). Alternatively, the increase in cortical neurons in *CR3* knockout mice could be driven by a signal emitted by mutant microglia that reduces the number of neurons that commit to apoptosis. Resolving these mechanisms will require precise measurement of the dynamics of apoptosis and microglial phagocytosis in the developing cortex.

In summary, we have presented evidence for the role of complement signaling in promoting the developmental elimination of neurons by microglia selectively in the ACC and shown that this process is required to achieve normal FC in adulthood. We failed to find evidence for a role of microglia complement signaling in synaptic or axonal pruning in the developing cortex. These findings call for further work to understand how neuronal apoptosis and phagocytosis can be regulated in a region-specific manner to shape adult brain connectivity and function.

## Supplementary Material

Slide4_bhad313

Slide5_bhad313

Slide6_bhad313

Slide7_bhad313

Slide8_bhad313

Slide9_bhad313

Slide10_bhad313

Slide11_bhad313

Slide12_bhad313

Slide13_bhad313

Slide14_bhad313

Slide15_bhad313

Material_and_Methods_03122022_for_proof_reading_bhad313
