# Supplementary figures and images for: Microglia complement signaling promotes neuronal elimination and normal brain functional connectivity
Source: Cereb Cortex. 2023 Sep 16;33(21):10750–60. doi: 10.1093/cercor/bhad313 (PMC10629900; doi:10.1093/cercor/bhad313)

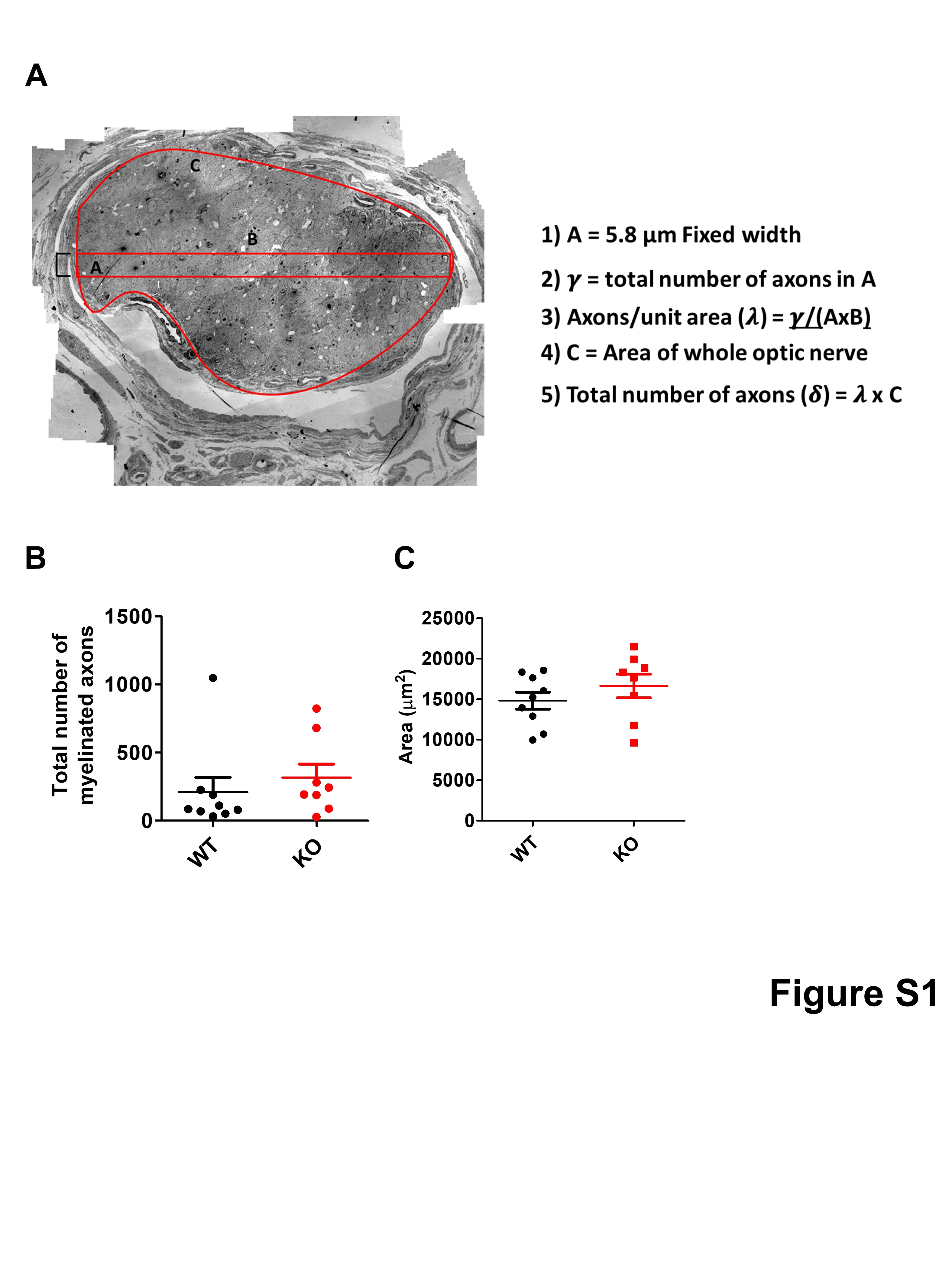

Supplement: Slide4_bhad313 [file slide4_bhad313.jpeg]

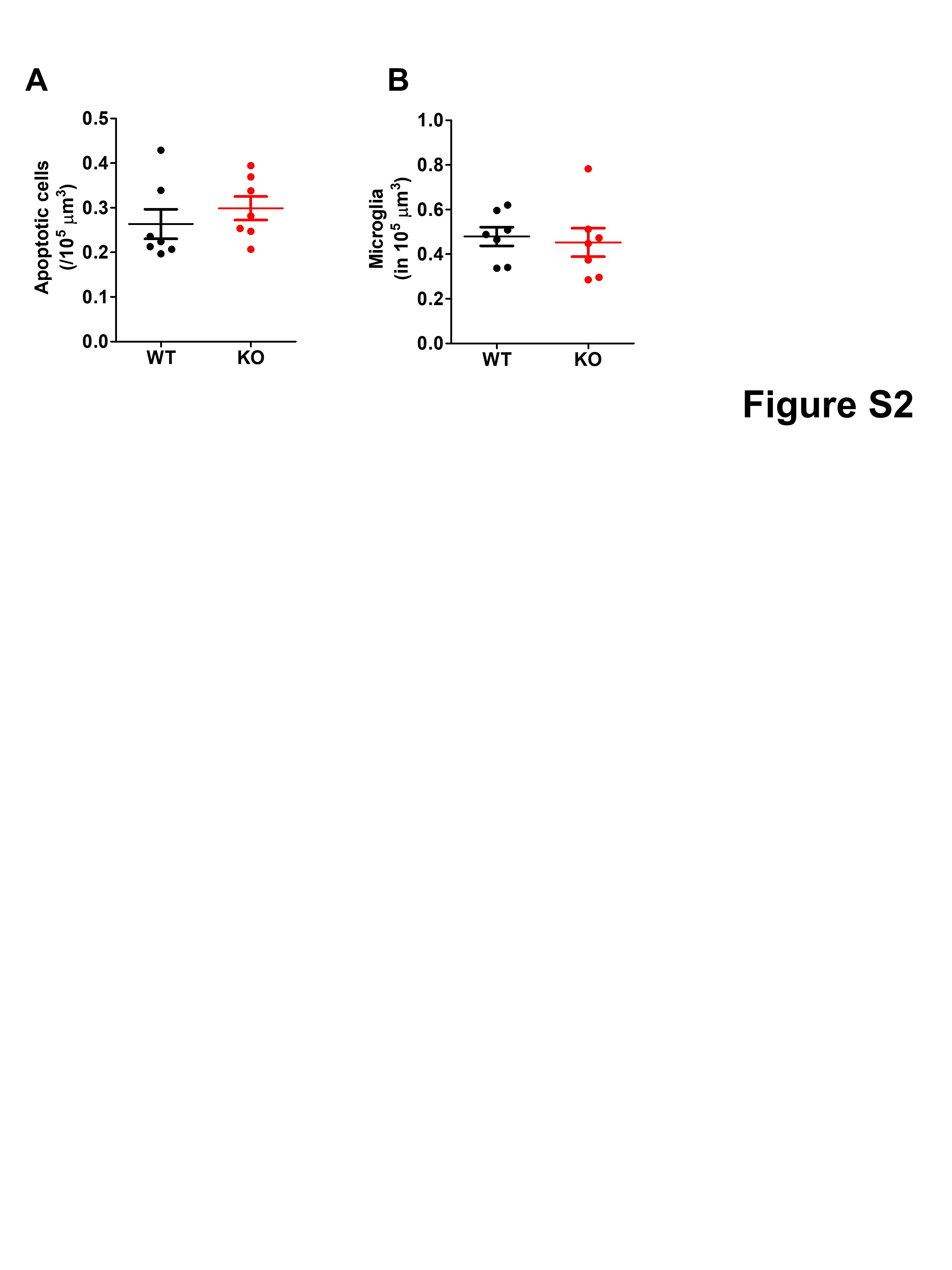

Supplement: Slide5_bhad313 [file slide5_bhad313.jpeg]

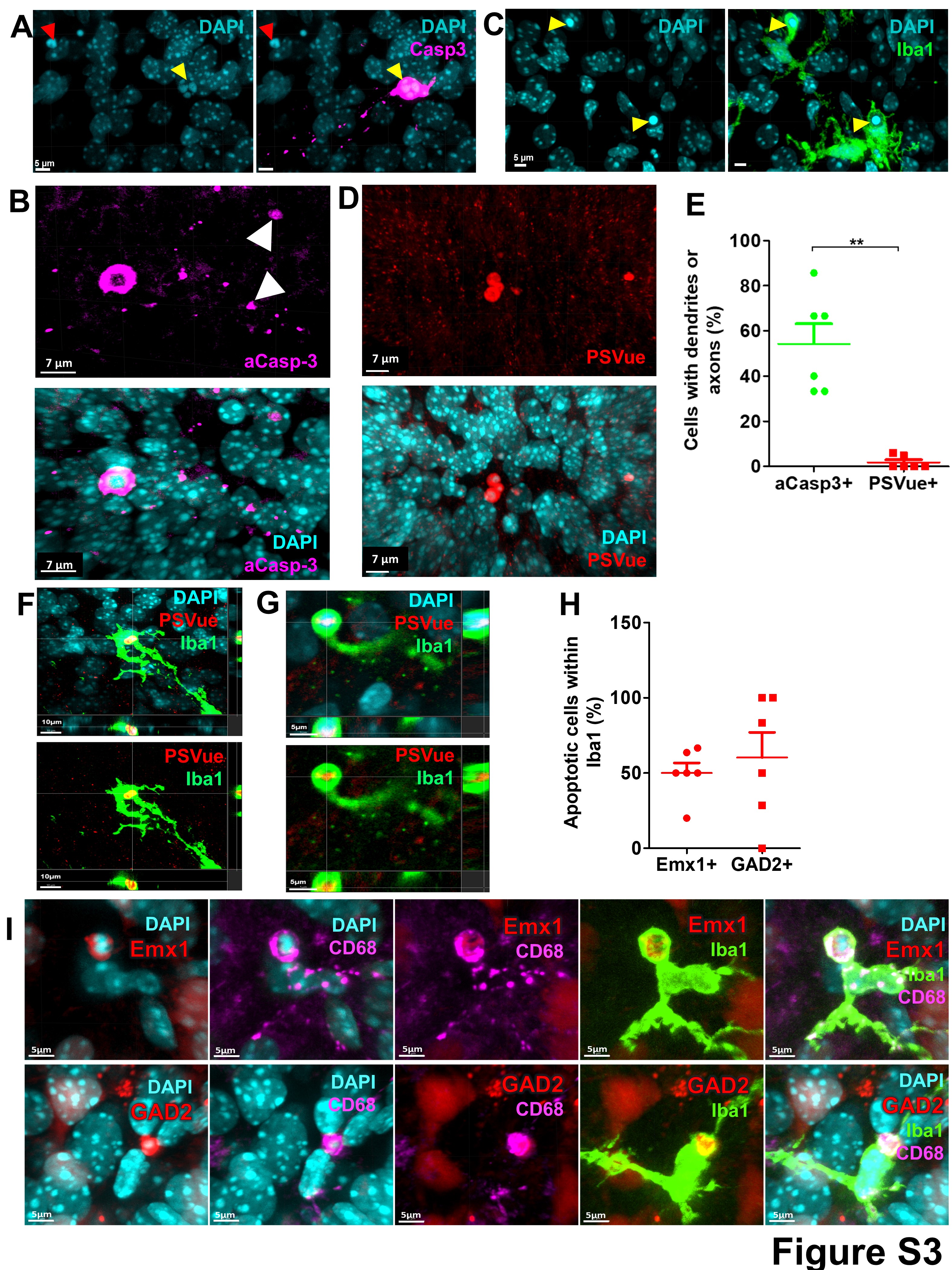

Supplement: Slide6_bhad313 [file slide6_bhad313.jpeg]

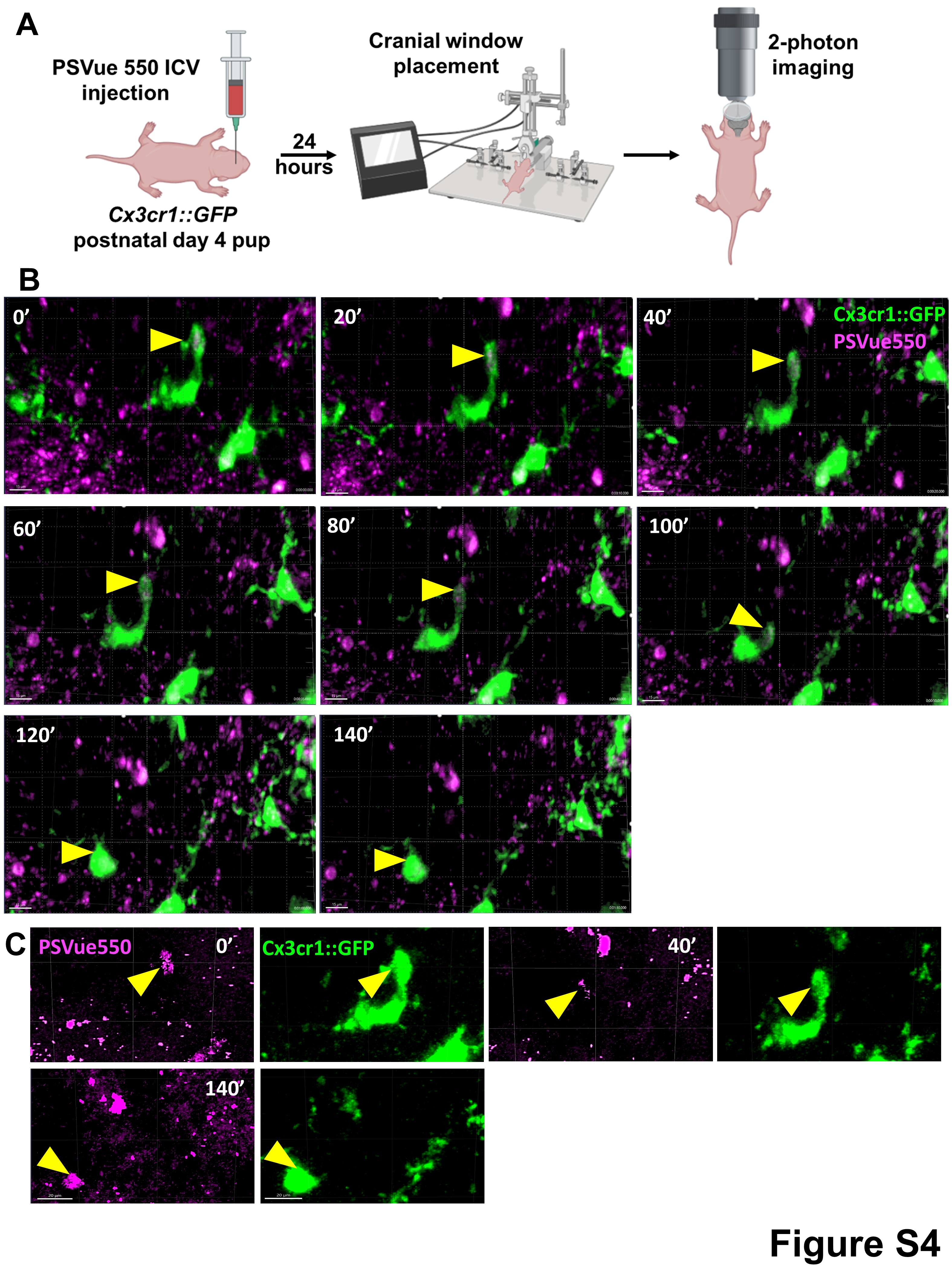

Supplement: Slide7_bhad313 [file slide7_bhad313.jpeg]

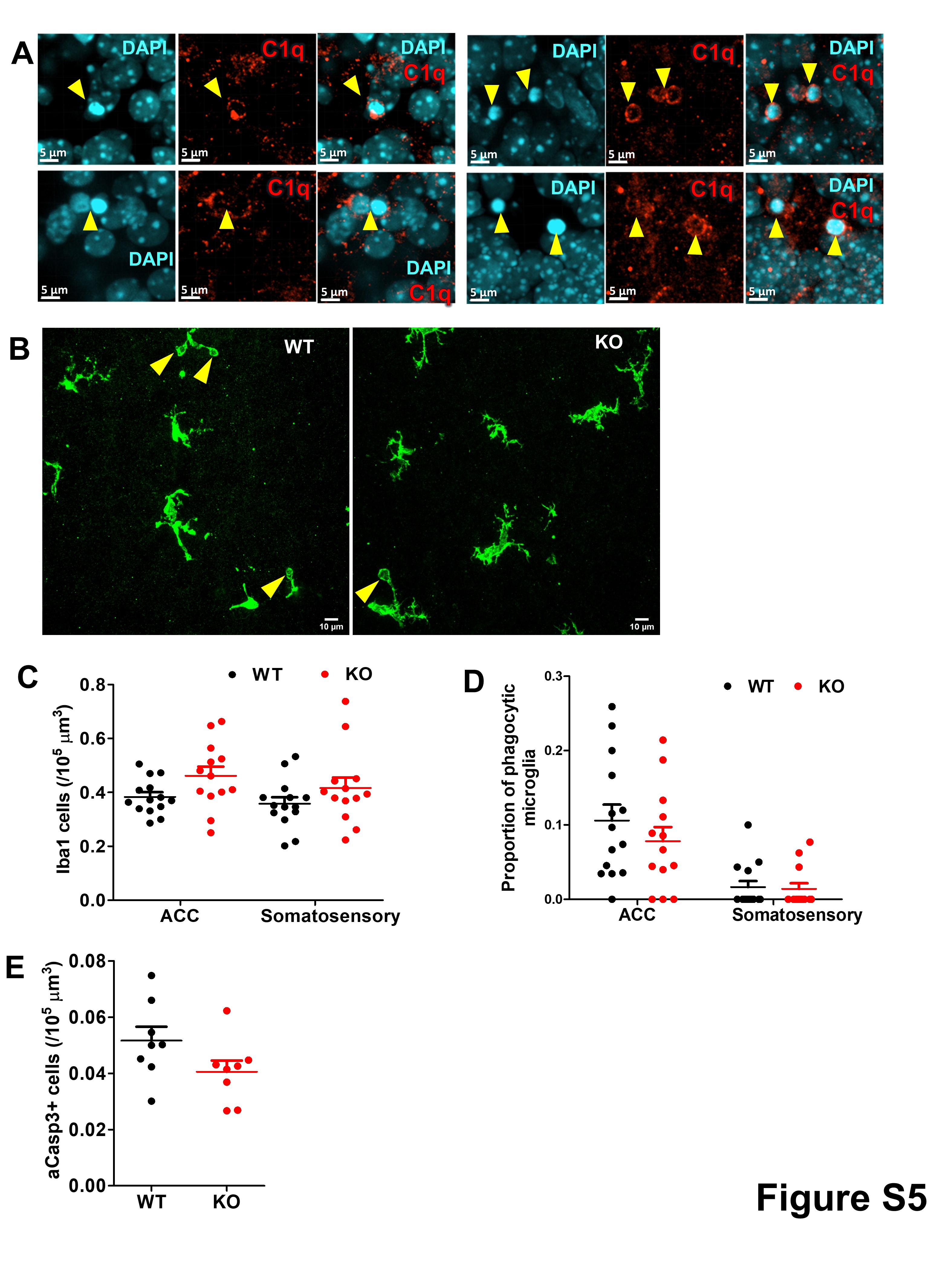

Supplement: Slide8_bhad313 [file slide8_bhad313.jpeg]

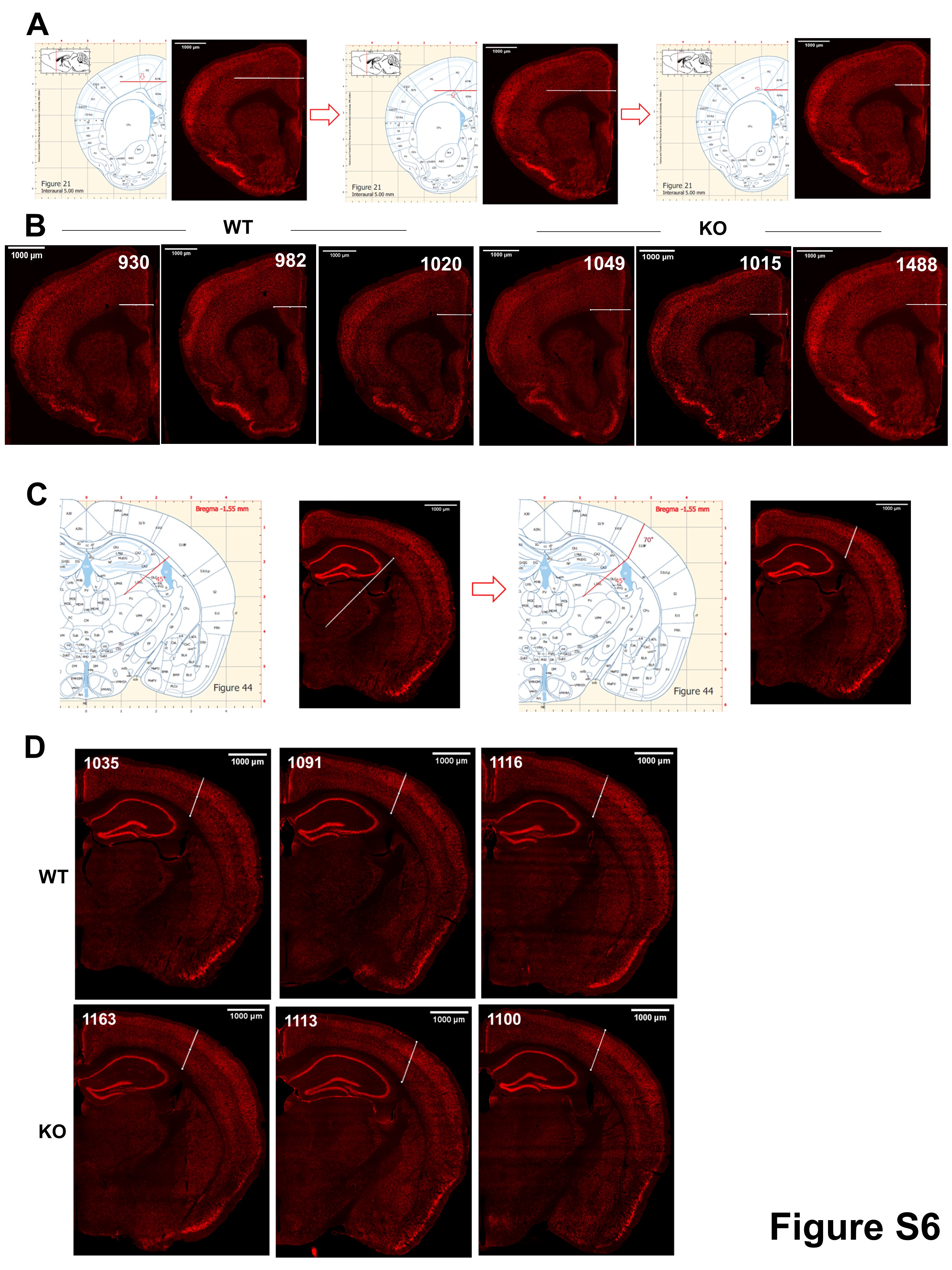

Supplement: Slide9_bhad313 [file slide9_bhad313.jpeg]

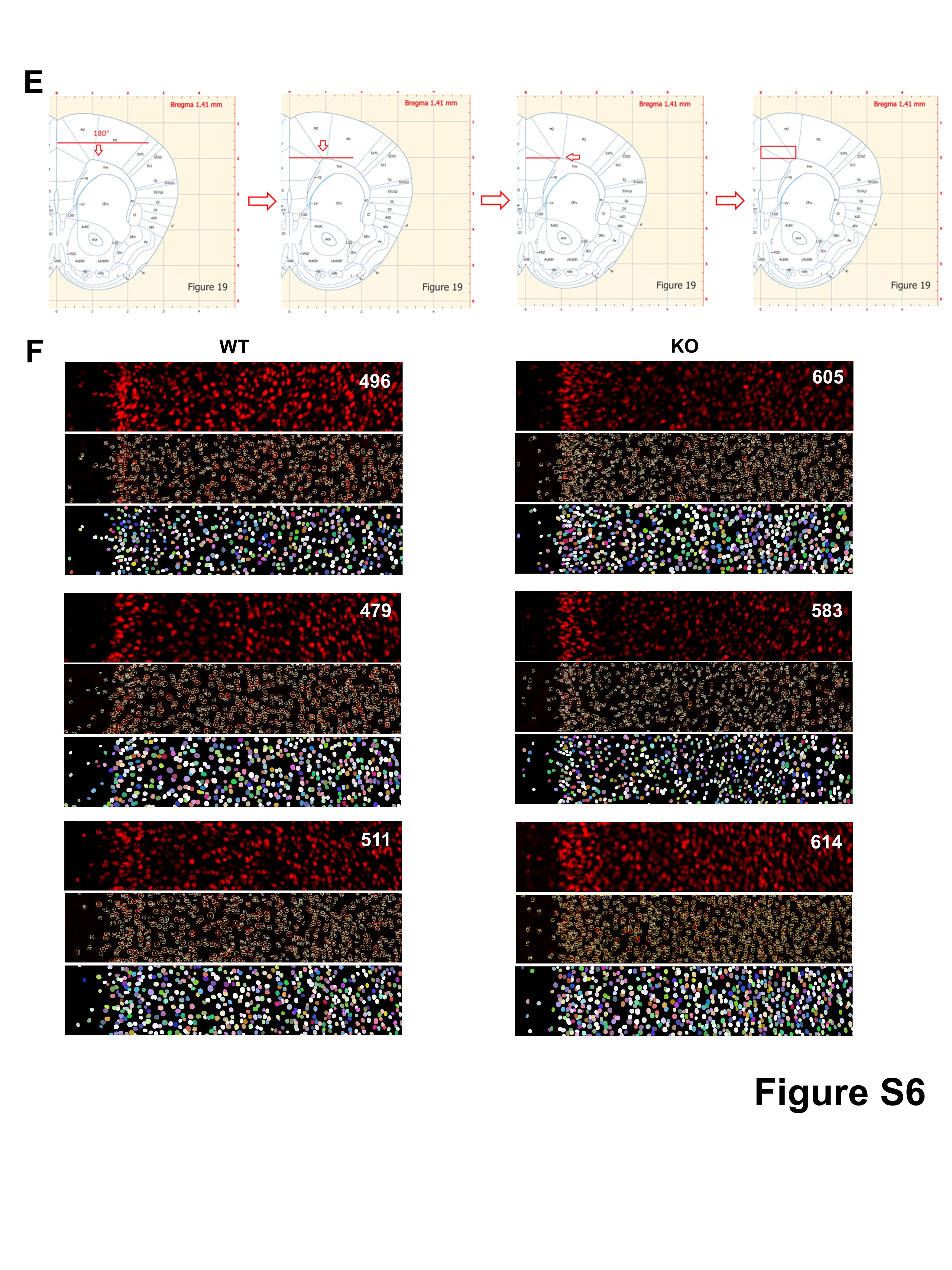

Supplement: Slide10_bhad313 [file slide10_bhad313.jpeg]

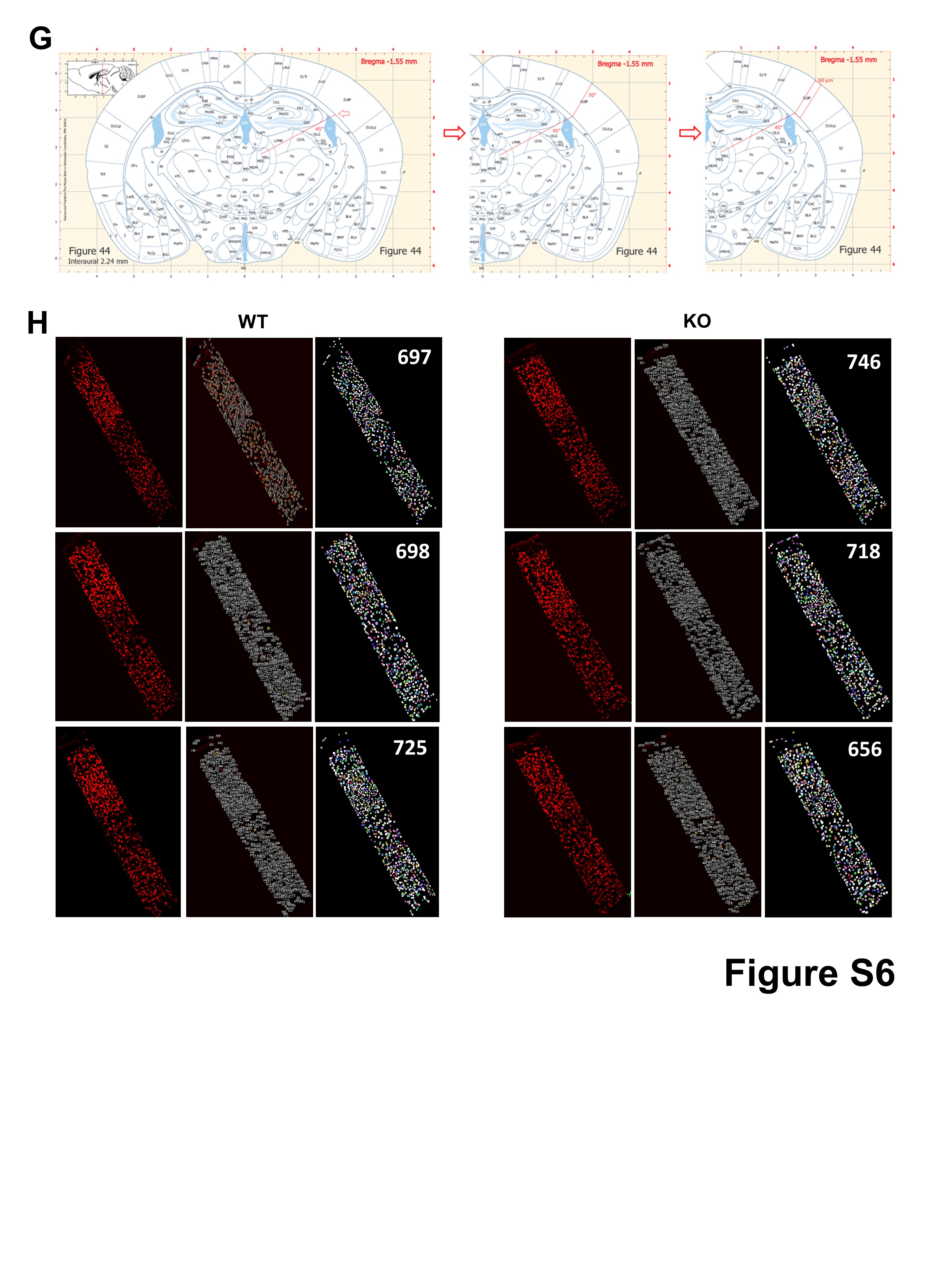

Supplement: Slide11_bhad313 [file slide11_bhad313.jpeg]

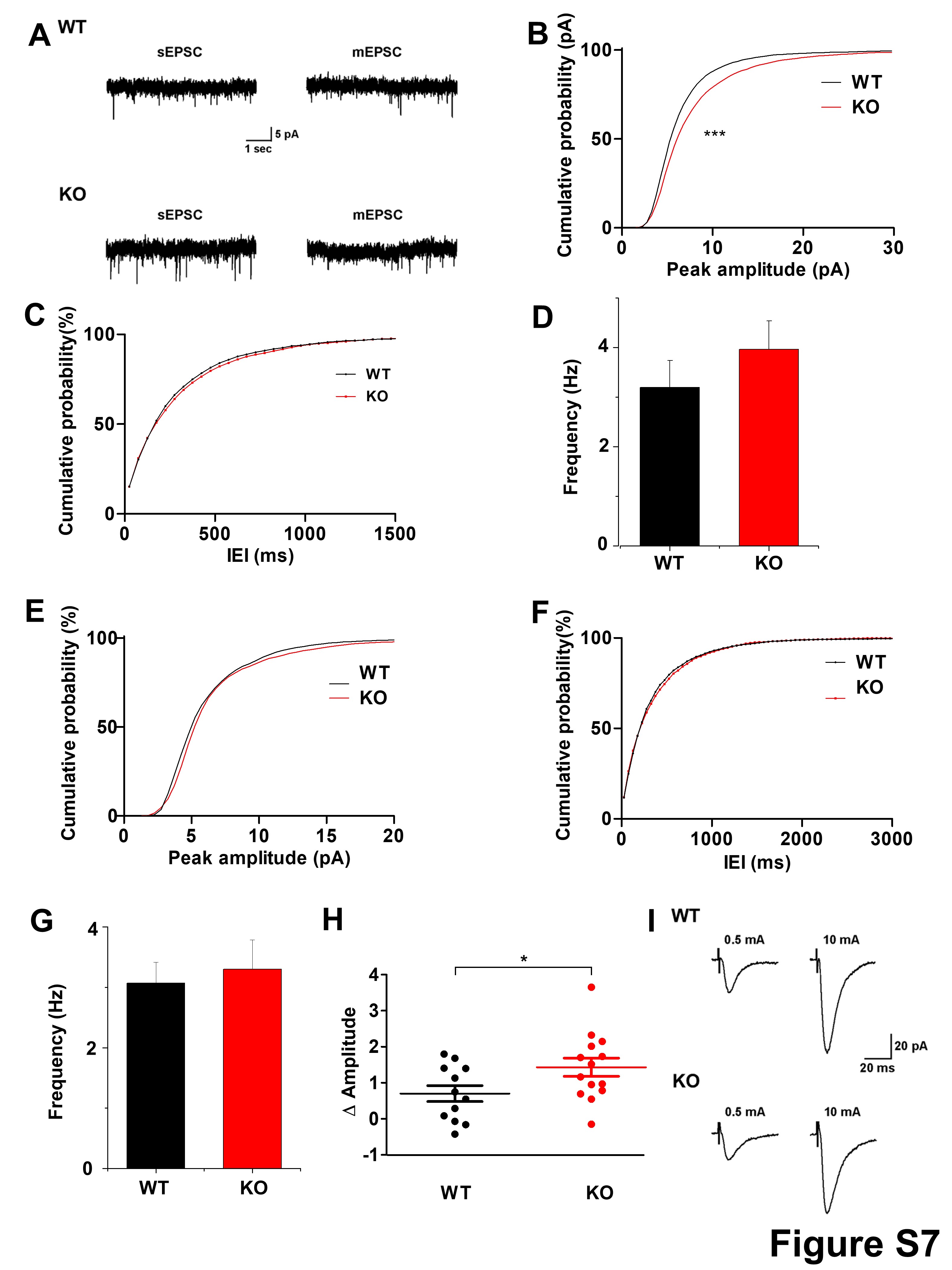

Supplement: Slide12_bhad313 [file slide12_bhad313.jpeg]

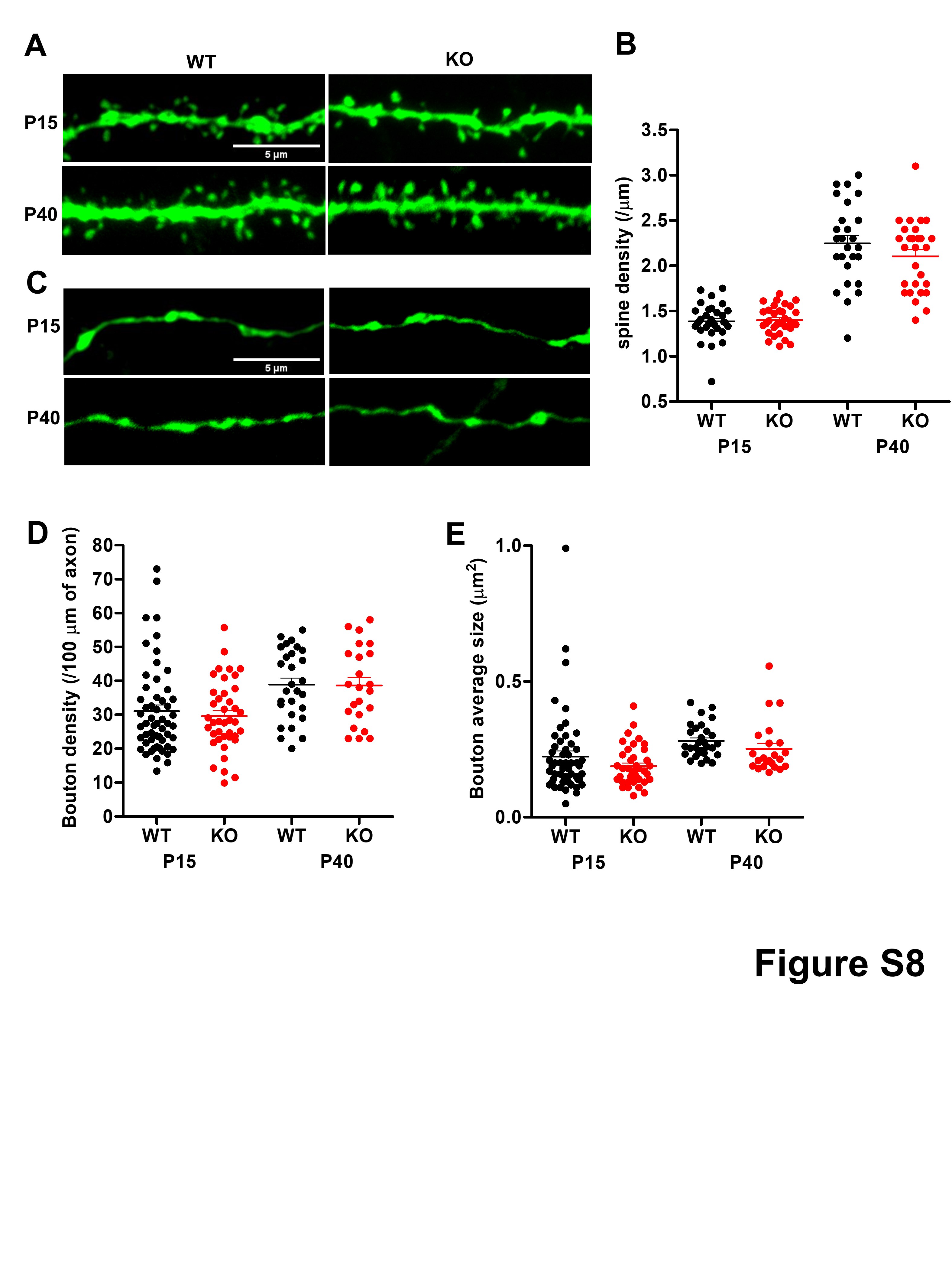

Supplement: Slide13_bhad313 [file slide13_bhad313.jpeg]

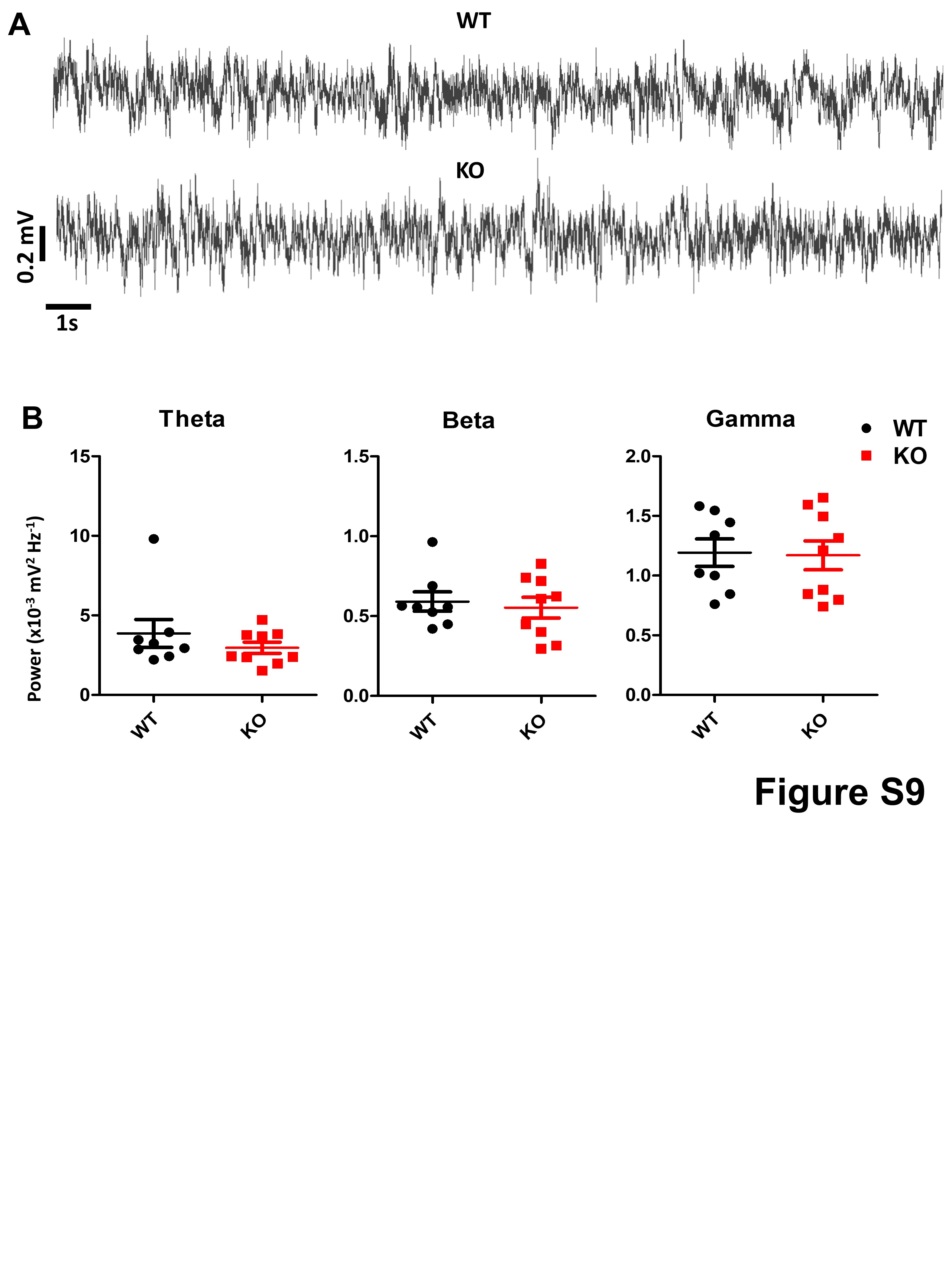

Supplement: Slide14_bhad313 [file slide14_bhad313.jpeg]

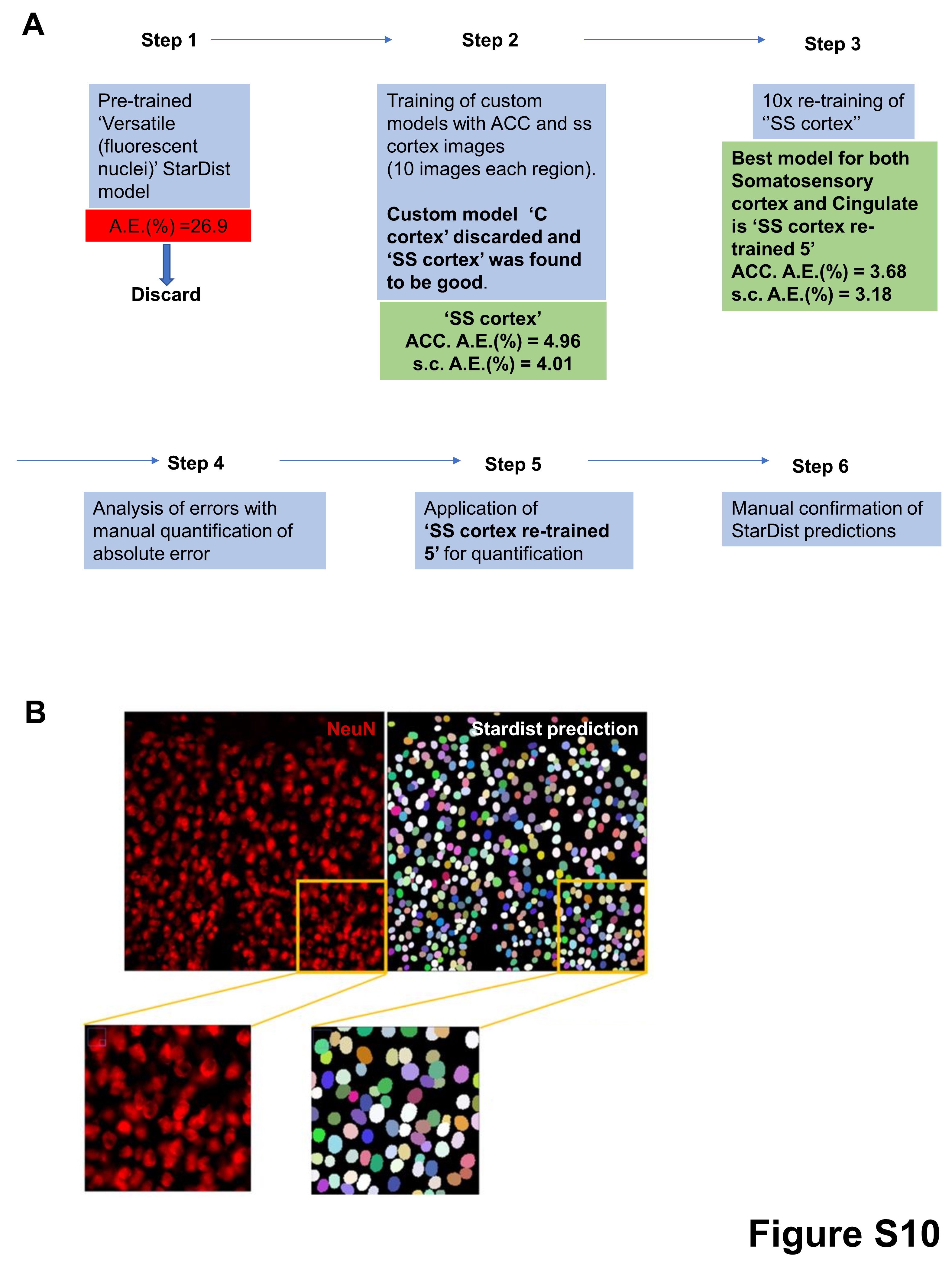

Supplement: Slide15_bhad313 [file slide15_bhad313.jpeg]
